# Impact of Beliefs About Local Physician Supply and Self-Rated Health on Willingness to See a Nurse Practitioner During the COVID-19 Pandemic: Web-Based Survey and Experiment

**DOI:** 10.2196/38965

**Published:** 2023-08-16

**Authors:** Celeste Campos-Castillo

**Affiliations:** 1 Department of Media and Information Michigan State University East Lansing, MI United States

**Keywords:** primary care shortage, workforce, health care seeking, public opinion, consumers, online studies, COVID-19, pandemic, primary care, nurse practitioners, nurse, healthcare, resources, advocacy

## Abstract

**Background:**

The COVID-19 pandemic overburdened primary care clinicians. For nurse practitioners (NPs) to alleviate the burden, the public must be willing to see an NP over a physician. Those with poor health tended to continue seeking care during the pandemic, suggesting that they may be willing to see an NP.

**Objective:**

The aim of this study is to evaluate the public’s willingness to see an NP for primary care and how this may be associated with their beliefs about the local supply of physicians and self-rated health. Two studies were conducted: (1) a survey to identify correlations and (2) an experiment to assess how willingness is dependent on information about the local supply of physicians.

**Methods:**

The survey and experiment were conducted digitally in April and December 2020, respectively. Participants were US adults recruited from Amazon’s Mechanical Turk platform. The key independent variables were self-rated health, which was a dichotomized 5-point scale (excellent, very good, good vs fair, and poor), and beliefs about local physician supply. The survey measured beliefs about local physician supply, while the experiment manipulated beliefs by altering information the participants read about the local supply of physicians. Willingness to see an NP was assessed in 2 ways. First as an overall preference over a physician and the second as a preference given 2 clinically significant scenarios in which participants imagined they were experiencing either coughing or a headache (presentation order randomized). Multiple regressions and ANOVAs were used to assess how beliefs about the local physician supply and self-rated health were associated with overall willingness to see an NP. Bivariate probits simultaneously estimated willingness to see an NP in the 2 clinically significant scenarios.

**Results:**

The survey showed that concerns about physician supply were associated with lower willingness to see an NP among respondents with comparatively better health but a greater willingness among respondents with comparatively worse health. The experiment suggests that only the latter is causal. For the 2 clinically significant scenarios, these patterns appeared for the coughing scenario in the survey and the headache scenario in the experiment.

**Conclusions:**

US adults with comparatively worse self-rated health become more willing to see an NP for primary care when they hear information that raises their concerns about the local physician supply. The differences between the survey and experiment results may be useful for interpreting findings from future studies. Findings may aid in managing finite health care resources during public health crises and crafting successful messaging by NP advocacy groups. Efforts to address nursing shortages will also be needed.

## Introduction

The COVID-19 pandemic imperiled an already fragile supply of US primary care clinicians [1-4]. To mitigate the risk of clinician burnout, medical errors, and long wait times for patients, several reforms were enacted in response, including relaxing guidelines for requiring physician supervision for nurse practitioners (NPs) [5,6]. The pandemic potentially created a window of opportunity for NPs to alter their practice conditions across the United States [5,7-10]. Successful long-lasting reform depends partly on the public’s willingness to seek care from NPs and thereby support their efforts [10-13]. In general, the public appears willing to see an NP, but during acute episodes, those with more severe symptoms prefer seeing a physician [11,12,14,15]. What remains unknown is whether the context of a public health emergency taxing the local physician supply may potentially alter these patterns, since patients who are concerned about wait times for seeing a physician and overburdening physicians are more willing to see an NP [11,12,15,16].

Another gap in the literature is that while a person’s overall health status is usually unrelated to their willingness to see an NP [12], it is unknown whether this may also change during a public health emergency. During the COVID-19 pandemic, health care visits dropped significantly but less so among those with poor health [17-19]. The potential for NPs to alleviate local physician supply shortages during a public health emergency may therefore be particularly strong among those with poor health since they are more likely to continue seeking care.

This paper adds to our understanding of how a public health emergency context shapes the public’s willingness to see an NP and whether this varies by overall health status. The paper summarizes the findings from 2 interrelated studies conducted to understand factors shaping the willingness of US adults to see an NP for primary care during the COVID-19 pandemic. The first is a cross-sectional survey designed to initially identify a correlation between a belief that the pandemic is overburdening local physician supply and a willingness to see an NP. The second is a randomized experiment that varied perceptions of the local physician supply to test whether information that raises concerns about the local physician supply affects willingness to see an NP. In both studies, analyses examined whether the relationship between concerns about the local physician supply and willingness to see an NP was contingent on respondents’ self-rated overall health. The findings will illuminate whether and how NPs can help address shortages in the local physician supply during public health emergencies such as the COVID-19 pandemic.

## Methods

### Ethics Approval

The institutional review board at the University of Wisconsin-Milwaukee approved procedures for both studies (#20.263). Prospective participants read the web-based consent form, which explained their enrollment in the study was voluntary and that they may skip any questions they did not wish to answer. Documentation of consent was waived, and prospective participants were informed while reading the web-based consent form that by proceeding beyond the form, they were consenting to their enrollment. Responses were deidentified by removing IP addresses from the data set. Participants were compensated with US $1 for their time.

### Setting

Both the cross-sectional survey and experiment were conducted digitally. The cross-sectional survey was conducted on April 8-9, 2020, and the experiment on December 8-10, 2020. Participants for both studies provided responses via a survey created using Qualtrics (Qualtrics), a web-based software that facilitates implementing several elements of the studies conducted, including randomizing the presentation order of similar questions and randomly assigning participants to an experimental condition.

### Participants

Respondents for both studies were recruited from workers on Mechanical Turk (MTurk) platform (Amazon). Compared to other convenience samples (eg, college students and social media users), MTurk respondents are more demographically diverse [20]. Only workers located within the United States were eligible for the study. To improve data quality, only workers with at least a 95% approval rating were invited to participate [21]. Furthermore, in order to be eligible to receive compensation, participants had to complete questions that assess the quality of their responses and steps to ensure they were located within the United States [22]. Workers who participated in the survey study were not eligible to participate in the experiment.

### Variables

#### Demographics

Both studies began by asking participants about their demographic background: sex, race and ethnicity, age (grouped as 18-34, 35-64, and 65 years or older to capture those who are eligible for Medicare), completing a college education, and having health insurance coverage. Participants in both studies were then asked about their prior experience with an NP. Participants read a description of NPs, which follows a definition used in other studies of NPs in the United States [12] and stated whether they had seen 1 previously.

#### Self-Rated Health

After providing their demographic background, both studies asked participants to rate their own health. Self-rated overall health was measured using a 5-point scale (1=poor, 5=excellent) and, consistent with other health research [23], transformed into a binary variable. The reference category for the analyses in both studies was participants with comparatively better health (1=poor, fair, good; 0=very good, excellent).

#### Concerns About Local Physician Supply

The point at which the 2 studies differed was after providing their self-rated health. In the cross-sectional survey, participants responded to questions measuring their concerns about the local physician supply and willingness to see an NP for primary care (the order in which these 2 were presented to participants was randomized within the survey). Conversely, participants in the experiment were randomly assigned to read a vignette describing the local physician supply and then responded to the same question as those in the survey that assessed their concerns about the local physician supply and willingness to see an NP. In this section, the vignettes presented to participants in the experiment are described first and then the question that assessed concerns. The items measuring willingness to see an NP are described in the next section.

The high supply vignette asked respondents to imagine the “local hospital receives their very first coronavirus patient. Over the next few days, the number of coronavirus patients they care for increases rapidly. The hospital announces they have more than enough physicians to care for all the patients who need medical attention.” In the low supply vignette, the hospital instead announces they “do not have enough physicians.”

Concerns about physician supply were measured on a 5-point scale (1=strongly disagree, 5=strongly agree) modified from COVID-19 pandemic surveys asking respondents’ agreement with the statement [24], “I feel that there are enough physicians in my community to handle the medical needs of the people who are seriously ill during the coronavirus outbreak.” For the analysis, the scale was reverse-coded, with higher values indicating stronger concerns about physician supply.

#### Willingness to See an NP

Items were modified from the Association of American Medical Colleges Consumer Survey. First, respondents were asked to “imagine [they] are searching for a new primary care provider. The practice [they] found has physicians and nurse practitioners that are all accepting new patients.” The respondents stated their overall preferences for seeing an NP on a 5-point scale (1=strongly prefer a physician, 5=strongly prefer an NP). Second, respondents were asked to imagine themselves in 2 clinically significant scenarios (the presentation order was randomized). The first read, “Imagine you have developed a worsening cough over the past several weeks and decide that you need to seek medical care. You call the clinic and are told you can see a nurse practitioner today or tomorrow, or you can see a physician in 3 days.” The second replaced “worsening cough” with “frequent severe headaches.” After each scenario, respondents reported their preferences for seeing an NP. As a conservative test of willingness to see an NP, respondents’ explicit preference for seeing an NP was categorized as a binary variable (1=prefer NP, 0=no preference or prefer physician).

### Study Size

The number of participants for the cross-sectional survey was 95 and for the experiment was 130. A power analysis using G*Power software (Heinrich Heine Universität Düsseldorf) showed that these sample sizes provide enough power (>.80) to detect a medium effect size (0.15) with a 2-tailed test using an α level of .05.

### Statistical Analyses

All steps were completed in Stata (version 16.1; StataCorp), and tests were 2-tailed. Preliminary analyses were conducted first to describe the samples for both studies and compare them to each other. Descriptive statistics (frequencies and percentages) were calculated to characterize both samples. *Z*-tests assessed how the characteristics of the experiment sample compared to the survey sample. For the experiment, the distribution of sample characteristics between the conditions was checked with chi-square tests. As a manipulation check for the experiment, a 2-sample *t* test compared responses to the concerns about physician supply between the 2 conditions.

The analyses for both studies examined how willingness to see an NP may vary by concerns about physician supply and self-rated health. The model specification, however, differed between the studies. For the survey, a linear regression estimated responses to the overall preference item, and a bivariate probit simultaneously estimated responses to the preference items that followed the 2 clinically significant scenarios. A bivariate probit estimates the likelihood of explicitly stating a preference for an NP in the 2 scenarios jointly, thereby accounting for dependencies between the 2 estimation equations and thus their correlated errors [25]. This accounts for observable (eg, having seen an NP previously) and unobservable (eg, actual local provider supply) similarities in the factors shaping explicit preferences in the scenarios. All regressions were estimated first without and then with an interaction term to assess whether the association between respondents’ concerns about physician supply and willingness to see an NP was contingent on their self-rated overall health. Estimates in all regressions were adjusted for covariates.

For the experiment, overall willingness to see an NP was analyzed using a 2-way ANOVA, and explicitly stating a preference for an NP in the 2 scenarios was analyzed using a bivariate probit. For both analyses, an interaction was estimated to assess whether the association between the assigned physician supply condition and willingness to see an NP varied by self-rated overall health. Unlike with the survey, respondents’ background characteristics were not included as covariates in the analyses. Doing so can introduce bias in the results from experiments designed to test interaction between manipulation and respondent characteristics [26].

## Results

All results for the survey are presented first, and then for the experiment. The majority of the survey respondents were male (n=62, 65%), White (n=65, 69%), college graduates (n=63, 66%), and between 35 and 64 years old (n=53, 56%). Most reported having health insurance coverage (n=90, 95%) and seeing an NP (n=63, 66.3%). Approximately half (n=50, 53%) reported comparatively worse self-rated overall health. The mean level of concern about physician supply was 2.6 (SD 1.2).

**Figure 1:**
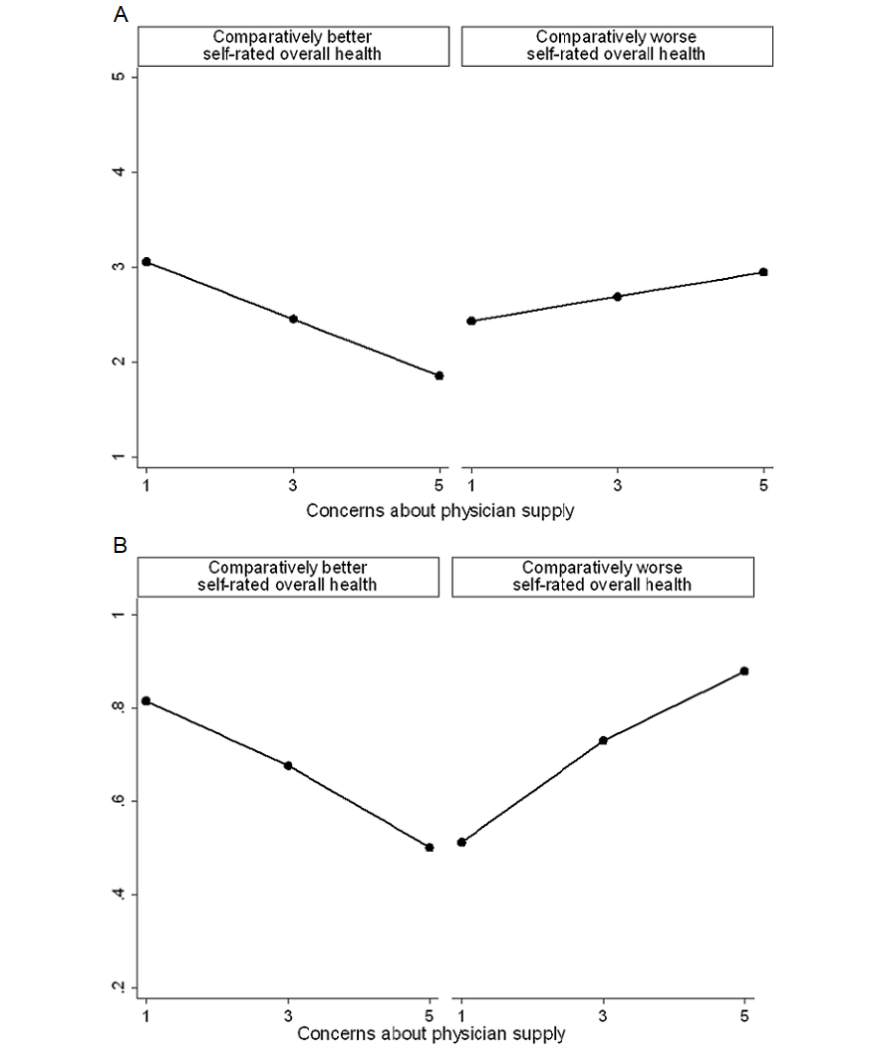
Willingness to see a nurse practitioner by self-rated health and concerns about local physician supply in survey. (A) Linear regression predicting overall preference. (B) Bivariate probit predicting explicit preference in coughing scenario.

Table 1 summarizes linear regressions predicting survey respondents’ overall preference to see an NP. Model 1 displays that neither concerns about the local physician supply (b=–0.112; *P*=.19) nor self-rated overall health (b=–0.342; *P*=.11) have significant adjusted associations with an overall preference for seeing an NP. Model 2 shows the results from adding an interaction between concerns about physician supply and self-rated overall health, which is statistically significant (b=0.396; *P*=.02) and displayed in Figure 1A.

Figure 1A plots the predictive margins with 95% CI, stratified by self-rated overall health. Among respondents with comparatively better self-rated overall health, concerns about physician supply are associated with lower overall preference to see an NP. Conversely, among those with comparatively worse self-rated overall health, concerns are associated with a greater overall preference.

Table 2 summarizes bivariate probit regressions performed on the responses from the survey respondents. The regressions analyze explicit preferences for seeing an NP for the 2 clinically significant scenarios. Model 1 shows explicit preferences are unrelated to concerns about physician supply (coughing: b=–0.010, *P*=.94; headache: b=–0.048, *P*=.69). Self-rated overall health is also unrelated to explicit preferences (coughing: b=–0.062, *P*=.82; headache: b=–0.201, *P*=.45). Model 2 displays results when estimating the interaction of interest, which is statistically significant for the coughing scenario (b=0.724, *P*=.01) and plotted in Figure 1B.

**Table 1:** Estimates from linear regression predicting overall preference for seeing a nurse practitioner (NP) in the survey.

Predictor			Model 1				Model 2		
			b (SE)		*P* value		b (SE)		*P* value
Concerns about local physician supply			–0.112 (0.086)		.19		–0.300 (0.114)		.01
Comparatively worse self-rated overall health			–0.342 (0.210)		.11		–1.377 (0.478)		.005
Concerns×comparatively worse health			N/A^a^		N/A		0.396 (0.165)		.02
Female (reference=male)			0.096 (0.224)		.67		0.133 (0.218)		.54
White (reference=minoritized racial or ethnic group)			–0.028 (0.234)		.90		–0.066 (0.228)		.77
**Age (years; reference=18-34 years)**									
	35-64	–0.429 (0.243)		.08		–0.518 (0.239)		.03	
	≥65	–0.639 (0.529)		.23		–0.594 (0.515)		.25	
College educated (reference=less than college)			0.324 (0.218)		.14		0.378 (0.213)		.08
Has health insurance (reference=uninsured)			0.873 (0.477)		.07		1.057 (0.471)		.03
Seen an NP (reference=never seen or do not know)			0.026 (0.233)		.91		0.067 (0.228)		.77

^a^N/A: not applicable.

**Table 2:** Estimates from bivariate probit predicting explicit preference for seeing a nurse practitioner for coughing and headache scenarios in the survey.

Predictor			Model 1										Model 2							
			Coughing					Headache					Coughing					Headache		
			b (SE)		*P* value		b (SE)			*P* value		b (SE)			*P* value		b (SE)			*P* value
Concerns about local physician supply			–0.010 (0.137)		.94		–0.048 (0.119)			.69		–0.316 (0.186)			.09		–0.091 (0.158)			.57
Comparatively worse self-rated overall health			0.003 (0.316)		.99		–0.184 (0.277)			.50		–1.950 (0.855)			.02		–0.454 (0.689)			.51
Concerns×comparatively worse health			N/A^a^		N/A		N/A			N/A		0.724 (0.294)			.01		0.098 (0.236)			.68
Female (reference=male)			–0.610 (0.330)		.06		–0.287 (0.291)			.32		–0.706 (0.340)			.04		–0.292 (0.290)			.31
White (reference=minoritized racial or ethnic group)			0.677 (0.345)		.05		0.475 (0.312)			.13		0.736 (0.352)			.04		0.484 (0.312)			.12
**Age (years; reference=18-34 years)**																				
	35-64	0.128 (0.338)		.70		0.249 (0.293)			.39		–0.006 (0.342)			.98		0.221 (0.299)			.46	
	≥65	–0.223 (0.810)		.78		0.384 (0.701)			.58		–0.147 (0.796)			.85		0.399 (0.698)			.57	
College educated (reference=less than college)			–0.082 (0.326)		.80		–0.168 (0.289)			.56		0.050 (0.332)			.88		–0.146 (0.290)			.59
Has health insurance (reference=uninsured)			1.460 (0.917)		.11		0.787 (0.831)			.34		1.780 (1.084)			.100		0.802 (0.829)			.33
Seen an NP (reference=never seen or do not know)			1.183 (0.326)		<.001		0.612 (0.296)			.04		1.440 (0.347)			<.001		0.636 (0.301)			.03

Figure 1B plots the predictive margins for the coughing scenario with 95% CI, stratified by self-rated overall health. Concerns about physician supply are associated with a lower likelihood of explicitly preferring an NP among those with comparatively better self-rated health but are associated with a greater likelihood among those with comparatively worse self-rated health.

The composition of the experiment sample was mostly comparable to the survey sample, with the majority being male (n=75, 58%), White (n=90, 69%), a college graduate (n=86, 66%), and between 35 and 64 years (n=70, 54%). Most reported having health insurance coverage (n=114, 87.7%) and seeing an NP (n=75, 66%). Approximately half (n=64, 49%) reported comparatively worse self-rated overall health. Two-tailed 1-sample *z* tests showed that significantly fewer people reported health insurance coverage than in the survey sample (*z*=–3.57; *P*<.001). No other characteristics differed significantly between the 2 samples. Nonsignificant chi-square tests indicate the distribution of the experiment sample’s characteristics between the 2 physician supply conditions was similar.

A 2-sample *t* test evaluated whether the information in the experimental manipulation succeeded in generating significantly different levels of concerns about the local physician supply. The 62 respondents in the low supply condition reported significantly greater concerns (mean 2.81, SD 1.29) than the 68 respondents in the high supply condition (mean 2.36, SD 1.27; t_128_=2.03; *P*=.04).

**Figure 2:**
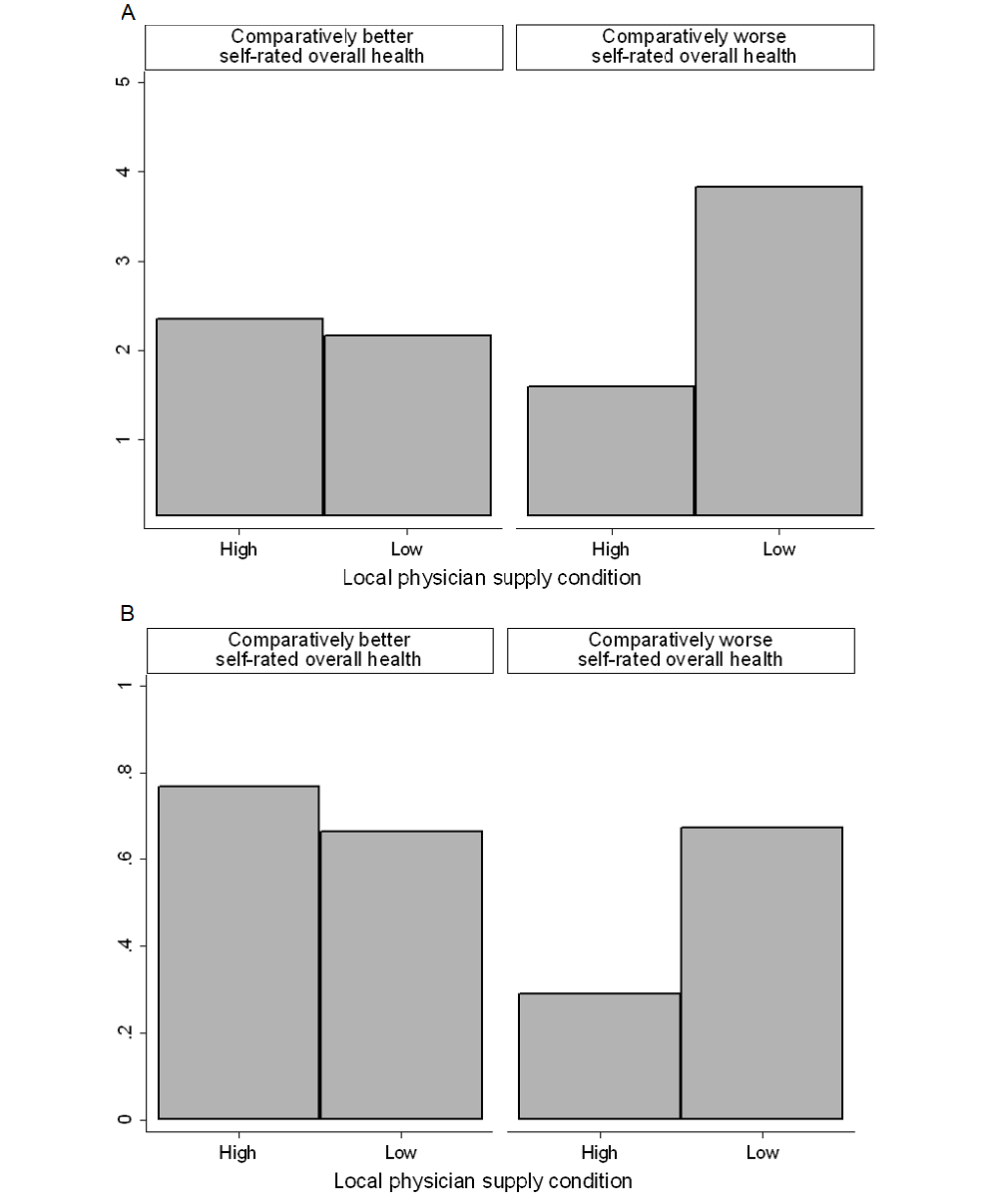
Willingness to see a nurse practitioner by self-rated health and assignment to physician supply condition in experiment. (A) ANOVA predicting overall preference. (B) Bivariate probit predicting explicit preference in headache scenario.

A 2-way ANOVA examined whether the overall preference for seeing an NP among respondents in the experiment was associated with their self-rated overall health and assignment to the local physician supply conditions. There was a statistically significant interaction between overall health and assigned condition as shown in Figure 2A (*F*_1,126_=8.96; *P*=.003). Among respondents with comparatively worse self-rated overall health, being assigned the low supply condition led to a greater overall preference to see an NP (mean 3.80, SD 0.84) than being assigned the high supply condition (mean 1.50, SD 0.71). Conversely, among respondents with comparatively better self-rated overall health, there were no significant differences in overall preference between the assigned condition (low supply: mean 2.17, SD 0.98; high supply: mean 2.36, SD 0.97).

Table 3 summarizes the bivariate probit regressions estimated within the experiment sample. The regressions analyze explicit preferences for seeing an NP for the 2 clinically significant scenarios. The first 2 columns show that neither self-rated overall health (coughing: b=0.195, *P*=.41; headache: b=–0.134, *P*=.57) nor assignment to the local physician supply conditions (coughing: b=–0.211, *P*=.38; headache: b=–0.192, *P*=.41) were significantly associated with preferences in the scenarios. The last 2 columns show estimates when adding an interaction between self-rated overall health and assignment to supply condition. The interaction is only statistically significant for the headache scenario (*b*=1.000; *P*=.03).

**Table 3:** Estimates from bivariate probit predicting explicit preference for seeing a nurse practitioner for coughing and headache scenarios in the experiment.

Predictor	Model 1				Model 2			
	Coughing		Headache		Coughing		Headache	
	b (SE)	*P* value	b (SE)	*P* value	b (SE)	*P* value	b (SE)	*P* value
Low local physician supply (reference=high local physician supply)	–0.211 (0.239)	.38	0.192 (0.235)	.41	–0.404 (0.330)	.22	–0.306 (0.332)	.36
Comparatively worse self-rated overall health	0.195 (0.240)	.41	–0.134 (0.234)	.57	0.006 (0.343)	.98	–0.607 (0.325)	.06
Low local physician supply×comparatively worse health	N/A^a^	N/A	N/A	N/A	0.392 (0.480)	.41	1.000 (0.472)	.03

^a^N/A: not applicable.

Figure 2B plots this interaction and shows assignment to condition only influenced preferences among respondents with comparatively worse self-rated overall health. Specifically, being assigned the low physician supply condition increased explicit preferences for seeing an NP.

## Discussion

### Summary of Findings

This paper summarized findings from 2 studies to evaluate how concerns about the local physician supply during the COVID-19 pandemic and self-rated health were associated with willingness to see an NP. Results from a cross-sectional survey showed that concerns about physician supply were associated with a greater willingness to see an NP among those with comparatively worse self-rated overall health but a lower willingness among those with comparatively better self-rated overall health. Moreover, these patterns appeared for a clinically significant scenario that asked respondents to imagine they were experiencing coughing. Because more negative ratings of self-rated overall health are associated with perceiving a less adequate supply of local physicians [27], the findings from the survey may be spurious. The experiment manipulated perceived physician supply to assess causality. Findings from the experiment showed that when the local physician supply appeared low, those with comparatively worse self-rated overall health are more willing to see an NP. For those with comparatively better self-rated overall health, their willingness is unrelated to the local physician supply. The patterns indicate the association between concerns about local physician supply and willingness to see NP is only causal among those with comparatively worse self-rated overall health. Notably, for the clinically significant scenarios, these patterns above appeared for only the headache scenario within the experiment. Conversely, in the cross-sectional survey, it was the coughing scenario that generated such patterns. The divergent findings may be due to the timing of the studies since the experiment was fielded after the Centers for Disease Control and Prevention added headache to the list of COVID-19 symptoms [28].

### Implications

The findings have important implications for managing the health care workforce supply. Compared to physicians, NPs are more likely to care for patients from vulnerable populations [29-31], and thus this study’s findings suggest that this difference may become amplified during crises such as the COVID-19 pandemic. Accordingly, it will be necessary to address ongoing nursing shortages and ensure there are sufficient NPs available with adequate training. As a means to encourage entry into the NP role, NPs will likely also need a workplace setting comparable to physicians, such as parity in insurance reimbursement and health care organizational policies facilitating admitting and other privileges. During the COVID-19 pandemic, NPs continued to lack these institutional supports in states enacting executive orders to expand their scope of practice [32,33]. Addressing these barriers will likely help encourage entry into the NP role and retention, thereby enabling NPs to contribute toward managing scarce health care resources.

By highlighting a shift in the public’s willingness to see an NP, this study can inform the advocacy efforts by NPs. Because those with poor health were more likely to continue seeking care during the COVID-19 pandemic [17-19], the findings from this study suggest NPs may target messaging toward this population. Such efforts may garner public support for full practice authority and overcome the practice restrictions that NPs face.

### Limitations and Future Research

Results should be interpreted in light of the design limitations. Because findings rely on reported intentions during hypothetical scenarios, additional work is needed to understand how best to translate intentions into actual help-seeking behavior. A difference in sample composition—fewer people reported health insurance coverage in the experiment—potentially contributed to the different patterns observed in the 2 studies. The difference is expected, given the losses in employer-sponsored health insurance coverage during the later periods of the pandemic [34]. Because health insurance coverage does not appear to drive health care seeking during the COVID-19 pandemic [35], it may not significantly shape other aspects of care seeking such as willingness to see an NP. Future research will need to directly examine how the loss of insurance coverage during a public health emergency may influence willingness to see an NP. The studies also cannot provide insight into whether any concerns about the quality of care or the expertise of NPs become attenuated during public health emergencies, including whether respondents would be more than just willing to see an NP but would trust the NP’s treatment recommendations and adhere to them. Previous research suggests patients with poor health may at times have lower levels of trust in providers [36]. Future studies should examine these topics, as well as how long patterns hold beyond the end of a public health emergency. Lastly, while relying on MTurk provides timely access to a diverse adult sample, important groups are underrepresented or missing, such as older adults and pediatric populations. Results should not be generalized to these groups.

### Conclusions

In conclusion, this study shows that US adults with comparatively worse self-rated health become more willing to see an NP for primary care when they read information stating a public health crisis such as the COVID-19 pandemic is taxing the local physician supply. Those with comparatively better self-rated health are less willing when they are concerned about the local physician supply, but this is unlikely causal. The combination of a cross-sectional survey and experiment is useful for highlighting issues that future cross-sectional studies should bear in mind. Moreover, findings may aid in managing finite health care resources during public health crises and crafting successful messaging by NP advocacy groups.

## Abbreviations

MTurk: Mechanical Turk
NP: nurse practitioner

## Data Availability

The data sets generated and analyzed during this study are available from the corresponding author on reasonable request.
